# Half-Century Scientometric Analysis: Unveiling the Excellence of Fungi as Biocontrol Agents and Biofertilisers

**DOI:** 10.3390/jof11020117

**Published:** 2025-02-04

**Authors:** Ziqi Yuan, Qi Shen, Kefei Yu, Yan Liu, Huabao Zheng, Yanlai Yao, Baolei Jia

**Affiliations:** 1 Key Laboratory of Soil Contamination Bioremediation of Zhejiang Province, College of Environmental and Resources Sciences, Zhejiang A&F University, Hangzhou 311300, China; yyuanzi77@163.com (Z.Y.); ykfoffice@zafu.edu.cn (K.Y.); 2 Xianghu Laboratory, Hangzhou 311300, China; liuyan@xhlab.ac.cn; 3 Institute of Environment, Resource, Soil and Fertilizers, Zhejiang Academy of Agricultural Sciences, Hangzhou 310021, China

**Keywords:** biocontrol agents, biofertilisers, bibliometric, arbuscular mycorrhizal fungi, *Trichoderma*, entomopathogenic fungi

## Abstract

Reducing the use of chemical inputs is becoming a major challenge in developing sustainable agriculture. Fungi, known as biocontrol agents (BCAs) and biofertilisers, are crucial in scientific research and are celebrated for their efficacy, eco-friendliness, and multifaceted roles. In this study, a bibliometric analysis was conducted on 5349 articles related to fungi as BCAs and biofertilisers over the past half-century using the Web of Science Core Collection (WoSCC) database. The publications on fungi, such as BCAs and biofertilisers, have increased significantly over the last 20 years, with a maximum growth rate of 33.7%. The USA and China lead in this field. Keyword clustering analysis revealed that entomopathogenic fungi, including Hemiptera, Coleoptera, and Lepidoptera, can be used to manage plant pests. It also showed that fungi can be used as biofertilisers to promote plant growth. The analysis of research trends shows that *Beauveria bassiana* in biological control is highly significant. This study also showed that entomopathogenic fungi control plant pests by infiltrating the insect cuticles. *Trichoderma* spp. exert biocontrol effects by producing antibiotics. Arbuscular mycorrhizal fungi can trigger plant defence mechanisms by modulating secondary metabolite synthesis. This study contributes to the current knowledge of fungi as BCAs and biofertilisers and can guide future research.

## 1. Introduction

To improve the performance of agricultural and food systems globally, crop pests and pathogens, barriers to food production, must be addressed [1]. Chemical pesticides and fertilisers play an essential role in satisfying the escalating global food demand; however, their application often has adverse effects, including soil fertility imbalances, acidification, and groundwater contamination [2,3]. Therefore, there is a need to develop alternative, cost-effective, and eco-friendly biocontrol agents (BCAs) and biofertilisers. These alternatives are essential for promoting sustainable agriculture, ensuring optimal nutrient uptake, and maintaining crop productivity [4,5].

Beneficial microorganisms, such as BCAs and biofertilisers, are promising sustainable alternatives to chemical treatments [1]. Microorganisms, including bacteria and fungi, have been developed over several decades to improve plant health and growth [3,6]. BCAs are favoured for their cost-effectiveness, efficiency, safety to beneficial organisms, environmental compatibility, low residual effects, and their role in fostering biodiversity [7,8,9,10]. Fungal BCAs have been widely applied because of their high reproductive rate, short generation time, and target specificity. Most biocontrol fungi are saprophytic, with *Trichoderma* exhibiting the most significant potential. *Trichoderma* has been extensively studied for its ability to inhibit soilborne pathogens and promote plant growth [11,12]. *Trichoderma virens* and *Trichoderma harzianum* are the most common commercially developed BCAs. They are particularly effective against root rot, fruit rot, damping-off, and wilt, which are often caused by pathogens such as *Pythium*, *Fusarium*, and *Rhizoctonia* [13,14].

Biofertilisers are substances that contain live microorganisms. When applied to seeds, plants, or soil, they colonise the rhizosphere or plant interior and promote its growth by increasing the nutritional availability for the host plant [4,15,16]. They boost crop yield, with potential increases ranging from 10−40%. Biofertilisers typically consist of bacteria or fungi that can fix nitrogen, solubilise phosphates, oxidise sulfur, produce hormones, or decompose organic compounds [5,17]. Beneficial fungi contribute to plant growth by producing siderophores, gluconase antagonists, antibiotics, and enzymes, such as cellulases and glycosidases, that degrade cell walls. The key plant growth-promoting fungi are *Penicillium, Trichoderma, Fusarium, and Phoma*. These fungi are non-pathogenic saprophytes that support crops by enhancing their growth and offering protection against diseases [18]. Additionally, *Glomus* spp., *Laccaria* spp., *Rhizoctonia solani*, *Glomus intraradices*, and *Paxillus involutus* can mobilise phosphates or enhance zinc solubilisation, thereby promoting plant growth [19,20,21,22]. Beneficial fungi contribute to plant growth by producing siderophores, gluconase antagonists, antibiotics, and enzymes that break down the cell walls of pathogens. The key plant growth-promoting fungi include *Trichoderma* and Arbuscular mycorrhizal fungi (AMF). Among these, *Trichoderma* is notable for its ability to release volatile organic compounds and dissolve soil phosphates, promoting plant growth [23,24,25]. Moreover, *Trichoderma* has been shown to effectively prevent and control soil-borne diseases in various crops, as well as certain leaf and ear diseases [26]. Similarly, numerous studies have demonstrated the role of AMF in enhancing the growth of crops such as watermelon seedlings, upland cotton (*Gossypium hirsutum* L.), and tomato [27,28,29]. Overall, fungi, such as BCAs and biofertilisers, are promising crucial tools for sustainable agriculture.

**Figure 1:**
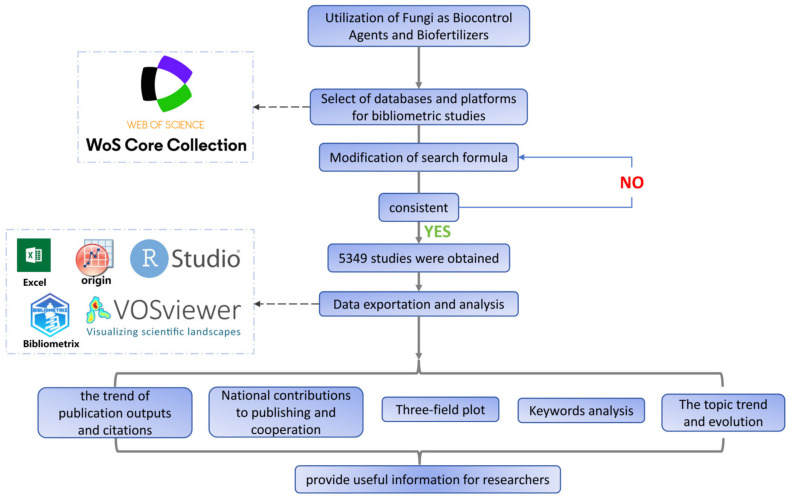
Comprehensive overview and flowchart of the study design.

Beneficial fungi reduce the impact of chemical pesticides and fertilisers on the environment and improve plant health, growth, and nutrient absorption [5,30]. Bibliometrics is a robust analytical tool for measuring scholarly literature. Bibliometrics tracks the progress of research fields and facilitates the identification of emerging trends and challenges (Figure 1) [31,32,33]. Data visualisation plays a key role in presenting analytical results and converting complex, large-scale bibliometric data into intuitive and interactive graphics for various audiences [34,35]. Visualisation also enhances the understanding and interpretation of bibliometric analyses by highlighting patterns, trends, clusters, gaps, outliers, and relationships within the data [36]. Using a comprehensive bibliometric analysis and literature review, this study seeks to reveal the current application status and research trends of fungi as BCAs and biofertilisers. We explored the potential of various fungi and elucidated their mechanisms of action in the field. We introduced strategies aimed at enhancing the efficacy of these applications. This study provides prospects for future developments in this field.

## 2. Materials and Methods

### 2.1. Data Collection and Processing

Web of Science Core Collection (WoSCC) database was used to search the literature in fields related to the application of fungi as BCAs and biofertilisers from 1976 to 2024. The specific retrieval formula was: Topic Search (TS) = (“fung*” OR “fung* extract” OR “fung* metaboli*” OR “fung* secondary metaboli*” OR “Beauveria bassiana” OR “ Metarhizium anisopliae” OR “Trichoderma” OR “Penicillium” OR “Arbuscular Mycorrhizal Fungi”) AND TS = (“bio*inoculant*” OR “bio-inoculant*” OR “biological inoculant*” OR “microb* inoculant*” OR “fung* inoculant*” OR “bio*fertiliz*” OR “bio-fertiliz*” OR “bio* organic fertiliz*” OR “bioorganic fertiliz*” OR “fungus fertilizer*” OR “fungal fertilizer*” OR “promote plant growth” OR “bio* control” OR “Biocontrol” OR “Bio*-control” OR “ Biological * control” OR “ Biolo* Management”) AND TS = (“plant disease resistance” OR “pest” OR “insect” OR “plant defense” OR “weed” OR “weedicide” OR “herbicide” OR “pesticide” OR “insecticide”) NOT TS = (Bacill*) NOT TS = (pseudom*) NOT TS = (plant growth-promoting rhizobac*).

### 2.2. Data Analysis

In this study, we used Excel 2021, Origin Pro 2021 to organise data. Bibliometrix 4.0 will serve as the primary tool for visualizing key metrics of each topic and the network linkages connecting them from various perspectives. VOSviewer 1.6.20 performs visual collinearity analysis on keywords.

## 3. Results

### 3.1. The Increasing Number of Publications Signifies That BCAs and Biofertilisers Have Emerged as Focal Research Topics

**Figure 2:**
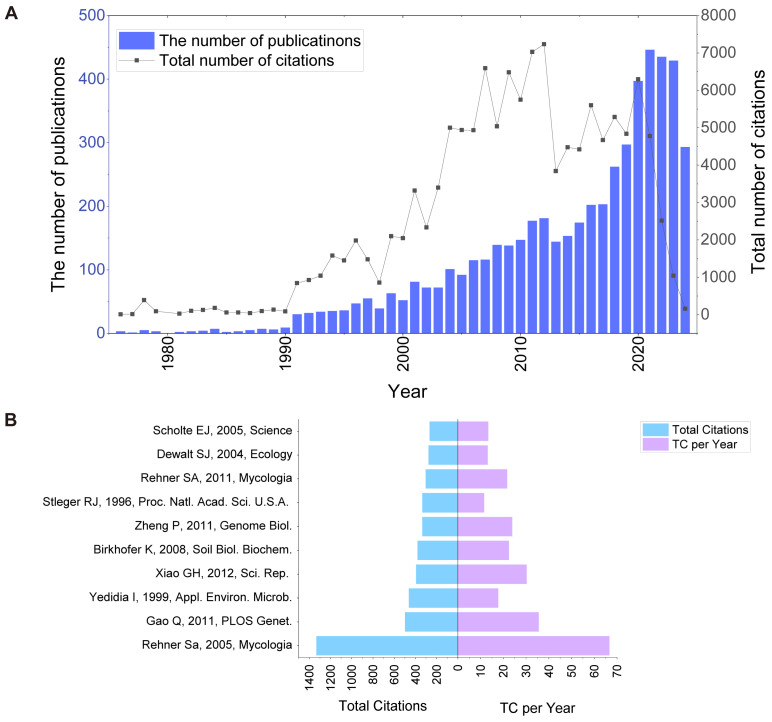
Literature publication and citations of fungi as BCAs and biofertilisers. (**A**) Number of publications on fungi as BCAs and biofertilisers and total citations per year, 1976–2024. (**B**) Total citations and average annual citations of the top ten most cited literatures on fungi as BCAs and biofertilisers [38,39,40,41,42,43,44,45,46,47].

The first article on BCAs and biofertilisers was published in 1976 [37]. We performed a bibliometric analysis of the literature in this domain from 1976 to 2024. As of July 2024, 5349 articles have been retrieved from the WoSCC database. Through extensive data analysis and visualisation of these papers, we found that publications related to the application of fungi as BCAs and biofertilisers showed different developmental periods (Figure 2A). Very few articles in this field were published between 1976 and 1990. There was a marked increase in publications from 1990 to 2016. The number of publications significantly increased from 2016 to 2021, with an average annual growth rate of 33.7%. Since 2021, the number of publications in this field has remained relatively constant. The citation analysis of publications from 2016 to 2021 showed that, although the publications have been cited for <10 years, the research results have a major impact. The total annual citations of these publications exceeded 4500, highlighting the influence of these research results in the field (Figure 2A).

We conducted a detailed analysis of the top 10 research papers with the highest citations to assess their influence within the field (Table 1) [38,39,40,41,42,43,44,45,46,47]. The first high-citation article established a phylogenetic framework that served as a basis for further taxonomic, phylogenetic, and comparative biological studies of *Beauveria* and their corresponding *Cordyceps* teleomorphs [38]. The second article presents a comparative analysis of the genome sequences of *Metarhizium anisopliae*, a broad-spectrum insect pathogen, and *M. acridum*, which specifically targets the acridids [39]. The third article explored the potential of *T. harzianum* T-203 to trigger plant defence responses by inoculating cucumber seedling roots in a hydroponic system [40]. This study demonstrated that *T. harzianum* penetrates the root system without causing extensive damage and transiently triggering host defence mechanisms. Other studies have primarily focused on the potential of insect pathogenic fungi for biocontrol, focusing on species such as *B. bassiana* and the *Cordyceps*/*Metarhizium* complex. Genome sequencing and genetic analyses were used to elucidate their potential roles (Figure 2B). *B. bassiana*, *M. anisopliae*, and *Cordyceps sinensis* (Isaria) are entomopathogens that affect soil-dwelling insects [48]. This prevalence could majorly contribute to their extensive research interest.

### 3.2. The USA and China Dominate the Field of BCAs and Biofertilisers

**Figure 3:**
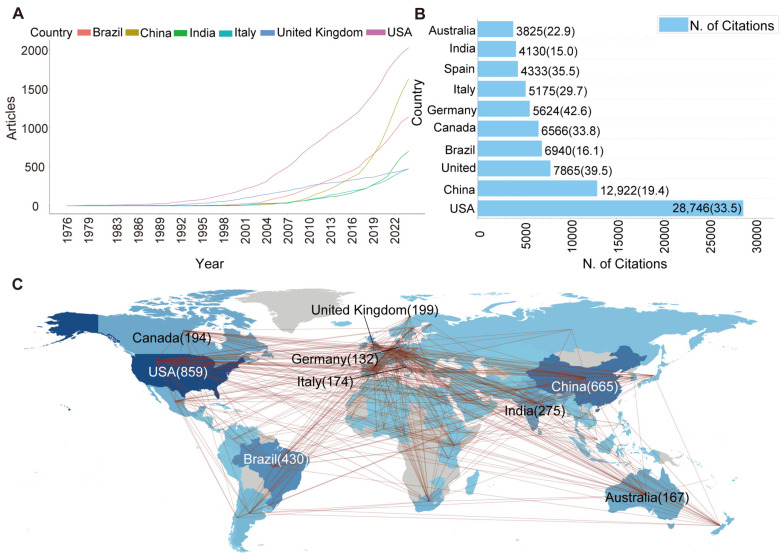
Number of publications on fungi as BCAs and biofertilisers in different countries and international cooperation. (**A**) The annual growth curve of article frequency for the top six countries on fungi as BCAs and biofertilisers from 1976 to 2024. (**B**) The top ten countries by total citation count. Numbers represent separately the total citations and the average article citations. (**C**) Global cooperation network in the field of fungal BCAs and biofertilisers. The intensity of the colour shades corresponds to the scientific productivity levels, with deeper colours indicating higher productivity. The numbers indicate the number of publications from each country.

Fungi have garnered global interest from scientists in BCAs and biofertilisers. From the annual growth curve of the frequency in each country (Figure 3A), the USA published its first paper in this field in 1977 [49]. The USA also has the most papers published in this field, reaching 859, indicating that it dominates the global scientific community. In 1996, China published its first English paper in the WoSCC database in this field in 1996 [50]. Despite starting later, China has recorded continuous growth in the number of published papers, ranking second after the USA, with 665 publications. In 2015, the Chinese government introduced the “Action Plan for Zero Growth of Fertilizer Use by 2020” and the “Action Plan for Zero Growth of Pesticide Use by 2020” [51]. These policies likely contributed to the exponential growth of publications by Chinese scientists, indicating that government initiatives played a key role in driving biofertiliser research.

The citations of publications in each country highlighted that the USA (28,746) and China (12,922) had more citations compared to other countries (Figure 3B). The USA accounts for half of the top ten highly cited documents, further showing the USA’s vital contribution to this field (Table 1). Among the top ten countries with the most citations, Germany ranked first regarding the average number of citations (42.6). This demonstrates that the results obtained by German researchers have significantly impacted this field.

The collaborative network confirmed that the application of fungi as BCAs and biofertilisers is a globally recognised field of research (Figure 3C). The USA partnered with nations, including China and Australia, on 132 journal publications, achieving an international collaboration rate of 15.4% (Figure S1). China co-authored 181 publications with authors from the United States, Canada, Brazil, and other countries, achieving an international collaboration rate of 27.2% and positioning itself as a major contributor in the field (Figure S1). Effective international cooperation plays an important role in promoting the development of this field and solving the challenges of sustainable agriculture.

### 3.3. Sankey Analysis Links the Institutes from Countries, Keywords, and Journals

**Figure 4:**
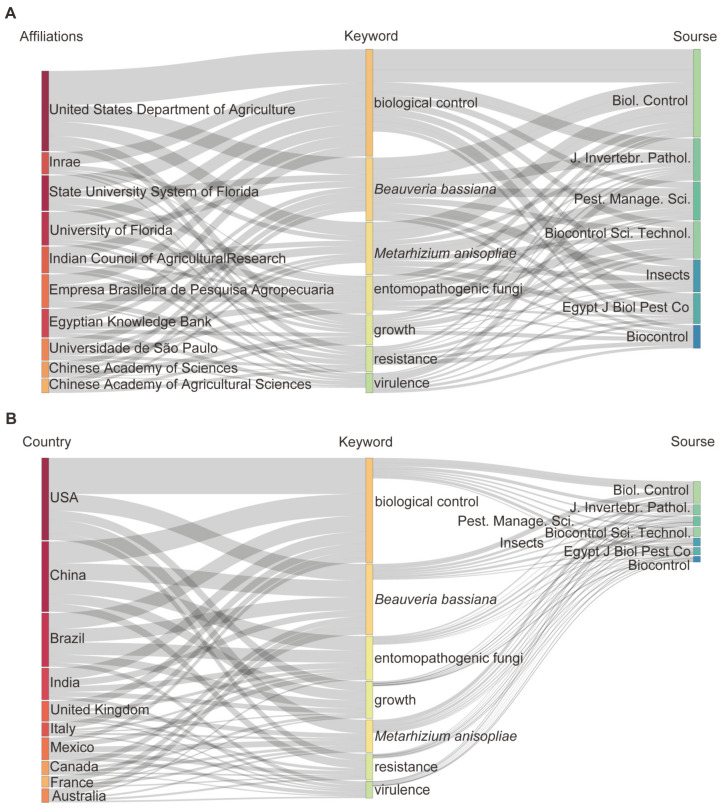
Sankey diagram showing the proportion of fungi as research subjects for BCAs and biofertilisers by affiliation and journal (**A**) and by country and journal (**B**) The sankey diagram consists of a series of “Nodes” and “Arcs”, that are read from left to right, with the thickness of each line proportional to the value it is representing.

Sankey diagrams offer an overview of the distribution of research topics across various institutions and countries and their preferred publication outlets, providing valuable insights into research trends and patterns (Figure 4). The results showed that the core research topics in the field included biocontrol, *B. bassiana*, *M. anisopliae*, entomopathogenic fungi (EPF), growth, resistance, and virulence (Figure 4A). Notable institutions contributing to this research include the United States Department of Agriculture (USDA), State University System of Florida, and University of Florida (Figure 4A). In addition, Biocontrol, Journal of Invertebrate Pathology, Pest Management Science, and Biocontrol Science and Technology were the preferred journals for publishing research on BCAs and biofertilisers (Figure S2). Overall, 14.8% of the journal articles were related to these fields (Figure S3). The aforementioned research institutions and academic journals encompass a comprehensive array of relevant subjects related to BCAs and biofertilisers (Figure 4A). The USDA predominantly publishes its research findings in renowned journals such as Biocontrol, Journal of Invertebrate Pathology, and Pest Management Science. The USA and China have led the field of biocontrol, focusing on the study of EPF, *B. bassiana*, and *M. anisopliae* (Figure 4B). The scholarly achievements of those nations are predominantly featured in biocontrol journals.

### 3.4. High-Frequency Keywords Show That B. bassiana and M. anisopliae Are the Key Fungi Used as BCAs and Biofertilisers

**Figure 5:**
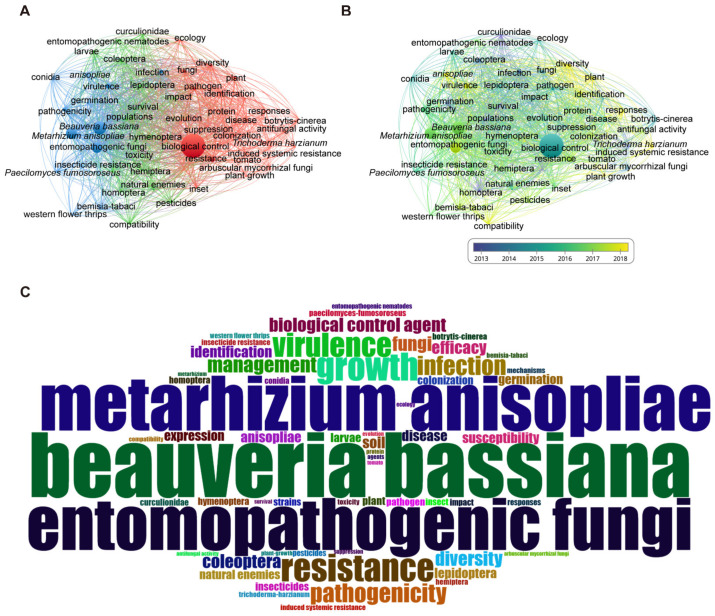
Keywords co-occurrence map on fungi as BCAs and biofertilisers. (**A**) Keywords co-occurrence network map. The blue sections illustrate the role of fungi as BCAs and biofertilisers in the comprehensive management of plant pests. The green section provides the types of pests that fungi can eliminate. The red sections of the analysis highlight the application of fungi as BCAs and biofertilisers in the effective control of plant diseases. (**B**) Co-occurrence network map of the year of occurrence (Frequency 100). Different colours correspond to different years. The closer the colour is to yellow, the more recent the year it represents. (**C**) Word cloud of keywords. The size of the font indicates the frequency of keyword occurrences. The larger the font, the higher the frequency. The circle indicates the frequency of keyword occurrence.

Keywords were used to describe the subject and main points of the study, with high-frequency keywords reflecting popular research topics. Visual analysis of keyword co-occurrence clustering highlights that biocontrol is a central focus in using fungi as BCAs and biofertilisers (Figure 5A,B). This study revealed three principal research trajectories in this field (Figure 5A). The first avenue focused on fungi as BCAs in managing plant pests. EPF, notably *B. bassiana* and *M. anisopliae*, are predominantly used to address the challenges posed by plant pests. The second research dimension mainly focused on the various insect pests that fungi can eliminate, such as Hemiptera, Coleoptera, and Lepidoptera. Research in these two areas was primarily conducted after 2016 (Figure 5B). The third research highlights fungi as BCAs and biofertilisers to combat plant diseases and promote plant growth, respectively. *Trichoderma* and AMF as BCAs are used to manage plant diseases and promote plant growth. Research on these two fungi was mainly conducted before 2016 (Figure 5B).

We used word clouds to conduct keyword mapping visualisations focusing on the most frequently occurring keywords. The word cloud prominently featured biocontrol and EPF, such as *M. anisopliae* and *B. bassiana*, highlighting their pivotal roles (Figure 5C). *B. bassiana* is an effective entomopathogen used to manage pests during red chilli cultivation [52]. It has several benefits, such as high insect mortality rates >85%, rapid colonisation, non-toxicity to treated plants, and plant growth enhancement. *M.* species have also been extensively studied and used as sustainable mycoinsecticides to control various pests [53,54].

### 3.5. The Evolution of Keywords Highlights B. bassiana as a Prominent Candidate for Future Biocontrol

**Figure 6:**
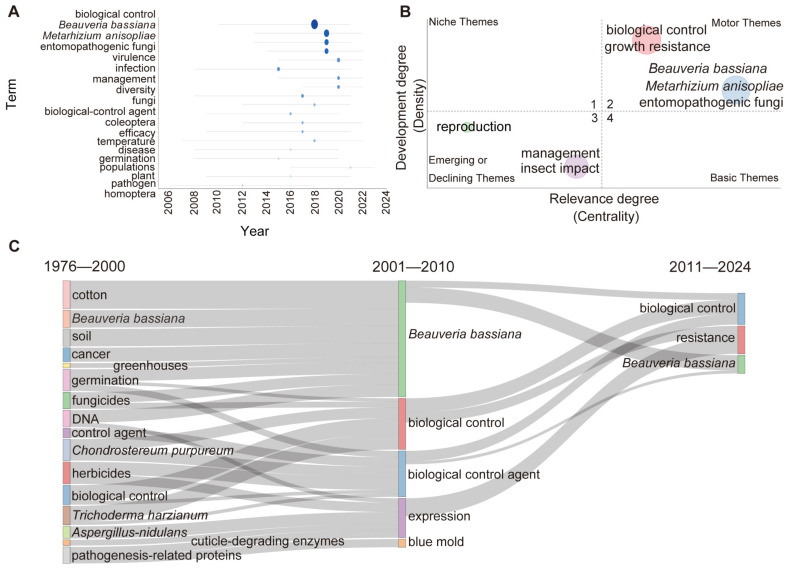
Trends on topics related to fungi as BCAs and biofertilisers. (**A**) The development trend of keywords with a frequency exceeding 100 occurrences. The size and colour intensity of the circles represent changes in frequency. Larger circles and darker colours indicate higher occurrence rates. (**B**) Thematic map of keywords. The horizontal axis represents the level of importance, while the vertical axis indicates the level of development. The first quadrant represents niche topics, characterised by low importance but high development. The second quadrant includes popular topics, which are highly important and well developed. The third quadrant represents declining topics with low importance and low development, while the fourth quadrant contains basic topics, which are of high importance but still in the early stages of development. (**C**) The thematic evolution of keywords in three stages. The length of the stripes represents the frequency of keyword occurrence. Longer stripes signify higher keyword frequencies during that period. The thickness of each line is directly proportional to the value it represents.

Analysing research trends enables researchers and practitioners to remain informed about the latest developments and align their work with the most relevant and current studies in the field. Our study explored the prospects of BCAs and biofertilisers by visually analysing the research trends of high-frequency keywords (Figure 6). The research findings showed a significant increase in the number and frequency of high-frequency themes related to the use of fungi in biological control and biofertilisers since 2016. From 2018 to 2020, the research focused mainly on biological control, emphasising EPFs such as *B. bassiana* and *M. anisopliae*. These highlight that these years have been pivotal for the expansion and in-depth exploration of this scientific field (Figure 6A). The core research topics in this field, biocontrol and insect pathogenic fungi with great development potential, such as *B. bassiana* and *M. anisopliae*, have become research hotspots and have been highly developed (Figure 6B). Growth resistance showed similar developmental trends. Basic topics such as reproduction, management, and insect impacts are in the later stages of research and are no longer currently researched extensively.

In addition, we visualised the development of keywords across three distinct periods (1976–2000, 2001–2010, and 2011–2024) using Sankey diagrams (Figure 6C). The keywords “*Beauveria bassiana*” and “biocontrol” appeared in all three stages, further confirming their importance in this field. In the first stage (1976–2000), the keywords were mainly basic keywords in this field and research on fungi, such as *Chondrostereum purpureum* and *Trichoderma harmzianum*. In the second stage (2001–2010), the research in this field focused mainly on *B. bassiana*. In addition to *B. bassiana*, biological controls, BCA, and their expression have also become research hotspots. The research focus of the third stage (2011–2024) was similar to that of the second stage (2001–2010), and further research was conducted on *B. bassiana* in biocontrol. Although *B. bassiana* and *M. anisopliae* are present in various ecosystems, *B. bassiana* tends to be dominant in natural habitats because it is susceptible to disturbances such as soil tillage and agricultural practices [55,56,57,58]. This may partly explain why research on *B. bassiana* is more prevalent than on *M. anisopliae*.

## 4. Discussion

As global food and environmental safety concerns intensify, agricultural sectors increasingly focus on using BCAs and biofertilisers. Beneficial microorganisms, particularly fungi, offer promising solutions to sustainable agriculture by enhancing crop growth and health and have blossomed over time. This section explores the species and mechanisms with the potential for biocontrol and plant growth promotion.

### 4.1. Species and Mechanisms of EPF with the Potential to Serve as BCAs

EPF are the high-frequency keywords that appeared in this study. EPF are highly effective natural pest enemies, making them valuable for biocontrol applications as environmentally friendly alternatives to chemical insecticides [59,60]. EPF can control various insect stages, from larvae to adults [61], and are known to infect all developmental stages of 20 out of 31 insect orders, including eggs, larvae, pupae, nymphs, and adults, being susceptible to infection [62,63]. *Lecanicillium* spp. are particularly important for managing small sucking insects such as aphids, thrips, whiteflies, and nematodes, especially in greenhouse settings [64,65]. *B. bassiana* is another well-known EPF used to control insect pests, such as whiteflies, thrips, mites, and aphids, across various developmental stages in numerous crops [66,67]. Using *B. bassiana* as a BCA offers several advantages, including high reproductive capacity, durable spore formation, environmental friendliness, and high pathogenicity against target pests [68]. Reportedly, *B. bassiana* effectively controlled *Aphis gossypii*, achieving an 80.00% mortality rate at a spore concentration of 10^6^ on the 4th day post-application [69].

**Figure 7:**
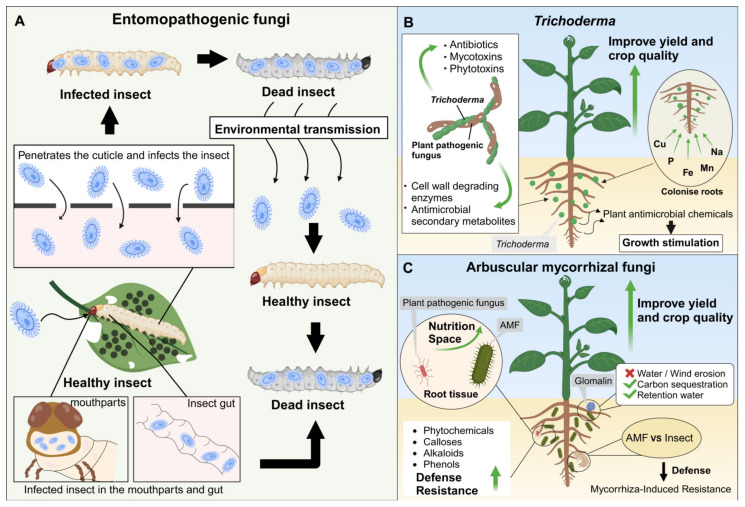
Mechanism of fungi as BCAs and biofertilisers. (**A**) Mechanism and effect of EPF as BCAs (insecticides). (**B**) The biocontrol mechanism and effect of *Trichoderma* in controlling plant pathogens and promoting crop health. (**C**) Mechanism and effect of AMF in controlling plant diseases and promoting plant growth. Figures created with BioRender.com.

Currently, more than 170 commercial products based on EPF are available, most of which contain *B. bassiana* and *M. anisopliae* [70]. Currently, there are about 750 species of fungi that can infect a wide range of insects and mites. EPF such as *Entomophtoromycota*, *Chytridiomycota*, *Ascomycota*, *Basidiomycota*, *Oomycetes,* and *Microsporidia* can infect and kill arthropods [71,72]. Unlike bacteria and viruses, which require ingestion to be effective, fungal BCAs directly penetrate the insect cuticle (Figure 7A) [73,74]. After penetration, the fungal spores germinate and form germ tubes that breach the cuticle, allowing them to enter the host. Upon invasion, spores proliferate and release toxins, eventually leading to death [75,76]. The infection rate depends on the fungal species and the number of spores involved [77]. Under favourable environmental conditions, the fungus emerges from the insect carcass, dispersing spores that can infect new hosts. Additionally, EPF secrete various extracellular enzymes that degrade insect cuticles, thereby facilitating infection [63,78]. Researchers have also observed other infection routes through studies on Oryctes larvae, desert locusts (*Schistocerca gregaria*), pine weevils (Hylobius pales), and Sitophilus granaries [79,80,81,82,83,84]. The conidia invaded the insect mouthparts and germinated in the insect gut (Figure 7A). Although fungal spores can adhere to the buccal cavity, the mechanism of insect death via spore ingestion remains largely unknown [70]. Further physiological and molecular studies are required to elucidate these underlying mechanisms.

### 4.2. Trichoderma Is a Key Fungal Species That Functions as BCAs and Biofertilisers

*Trichoderma* was another high-frequency keyword used in our study. The genus *Trichoderma* has garnered considerable attention because of its beneficial role and can serve as BCAs and biofertilisers (Figure 7B). Approximately 50−60% of fungal BCAs belong to this genus, and the European Union has approved approximately 77 commercial biofungicides based on *Trichoderma* [13,85]. Notable species with promising biocontrol potential include *T. harzianum*, *T. hamatum*, *T. asperellum*, *T. viride*, *T. koningii*, *T. pseudokoningii*, *T. afroharzianum*, and *T. cyanodichotomus* [86]. Research on *T. harzianum* was extensive before 2000, with a gradual shift towards its role in biocontrol from 2000 to 2010 (Figure 6C). *T. viride* is particularly effective against soil-borne pathogens such as *Fusarium*, *Sclerotium*, *Rhizoctonia*, and *Pythium* [3]. Various rhizospheric and epiphytic *Trichoderma* species are used in products because they can mitigate abiotic and biotic stresses in host plants by controlling a wide range of pathogens and nematodes [67]. Integrating *T. guizhouense* NJAU 4742 into bio-organic fertilisers augmented cucumber production in field settings [87,88]. The growth-promoting properties of *Trichoderma* extend to various crops, including soybean [89], cotton [90], sugarcane [91], and rice [92].

*Trichoderma* employs various mechanisms to exert biocontrol, including mycoparasitism, secondary antibiotic metabolite production, nutrients and space competition, and systemic immune response induction in plants [93,94,95] (Figure 7B). Mycoparasitism is a key biological control strategy employed by *Trichoderma* spp. to detect and degrade phytopathogens [96]. *Trichoderma* releases various cell-wall-degrading enzymes (CWDEs) through contact with plant pathogens, which break down the structural components of the phytopathogen’s cells [93,97]. *Trichoderma* strains produce low-molecular-weight volatile or non-volatile antibiotics or diffusible compounds that interact with and restrict the growth of deleterious plant pathogenic fungi in a process called antibiosis. Metabolites produced by *Trichoderma*, including antibiotics, mycotoxins, and phytotoxins, play crucial roles in antagonism through mechanisms such as antibiosis, competition, and hyperparasitism. The fungus secretes enzymes such as glucanases, chitobiosidases, and chitinases with antibiotics such as viridin, gliotoxin, and peptaibols, further contributing to its antagonistic capabilities [98]. *Trichoderma* generates many secondary metabolites, including trichodermin, gliotoxin, viridin, and peptide-based antibiotics [99]. Antibiosis is crucial in managing *Pythium ultimum* and *Rhizoctonia solani*, which cause damping-off in zinnias. Gliotoxin, produced by the BCA *Gliocladium virens*, inhibits the growth of *R. solani* and *P. ultimum* by disrupting membrane integrity and causing metabolite leakage [100]. Furthermore, *Trichoderma* employs a dual strategy involving CWDEs and antimicrobial secondary metabolites to counteract its host, thereby facilitating the formation of attachment and infection structures [100].

The rhizosphere competence of *Trichoderma* enables it to colonise root surfaces, effectively competing with other microorganisms for root-secreted nutrients in rhizospheric soil [101]. *Trichoderma* spp. often outcompete other microorganisms in the soil because they can mobilise and absorb essential nutrients such as copper (Cu), phosphorus (P), iron (Fe), manganese (Mn), and sodium (Na) [102] (Figure 7B). *Trichoderma*’s root colonisation further enhances plant growth, development, and stress tolerance. In addition, *Trichoderma* forms mutualistic associations with the rhizosphere, naturally enhancing plant nutrition, growth, and abiotic stress resistance [103]. Notably, antimicrobial compounds produced by *Trichoderma* can also stimulate plant growth, highlighting its multifaceted role in promoting plant health [100,104] (Figure 7B).

### 4.3. Mechanisms of Mycorrhizal Fungi with the Potential to Serve as BCAs and Biofertilisers

Mycorrhizal fungi also appeared frequently in the articles analysed in this study. Mycorrhizal fungi contribute to abiotic stress reduction and play a role in biocontrol by suppressing root-damaging pathogens, including nematodes and species from the genera *Fusarium*, *Pythium*, and *Phytophthora* [105,106]. Among mycorrhizal fungi, AMF are the most prevalent, producing the largest biomass and being beneficial to plants. AMF possess well-documented antagonistic and inhibitory effects on soil-borne pathogens [107,108]. Over 30 AMF species have proven effective in controlling plant diseases caused by these pathogens [43,109]. Mycorrhizal fungi are widely used as biofertilisers because they can extend the root systems of host plants, thereby facilitating water and nutrient uptake, particularly phosphorus. Field trials have demonstrated that mycorrhizal inoculation can increase crop yields, such as potatoes [110], maise [111], and yams [112], indicating that mycorrhizal fungi can directly enhance crop yield and quality. Numerous biocontrol and biofertiliser products based on mycorrhizal fungi, particularly strains of *Glomus iranicum*, enhance crop water and nutrient uptake and increase nematode tolerance. Other mycorrhizal inoculants, including *Rhizophagus irregularis*, *Funneliformis mossae*, and *Claroideoglomus etunicatum*, improve fruit production [113]. In addition to their direct benefits in plants, AMF indirectly enhances soil characteristics, promoting plant growth (Figure 7C). AMF improves soil resistance to erosion by wind and water through soil structure enhancement, facilitated by glomalin production, a glycoprotein that aids soil carbon sequestration [114,115,116]. Glomalin promotes water retention within the soil due to its positive effect on soil structure [117].

Studies have demonstrated that AMF regulates secondary metabolite production in host plants through multiple mechanisms. These mechanisms include altering the morphology and anatomical structure of plant roots and enhancing the physical and chemical properties of the rhizosphere. These mechanisms also include They also encompass competition with pathogens for photosynthetic resources and infection space, improving photosynthesis and nutrient uptake, and activating plant disease resistance and defence systems. These mechanisms include altering the morphology and anatomical structure of plant roots, enhancing the physical and chemical properties of the rhizosphere, competing with pathogens for photosynthetic resources and infection space, improving photosynthesis and nutrient uptake, and activating plant disease resistance and defense systems [118,119,120,121] (Figure 7C). AMF produces compounds such as phytochemicals, calluses, alkaloids, and phenols on the surfaces of inner and outer root hyphae, which help plants withstand disease-induced stress [122]. *Glomus mosseae* induces a phytotoxin stress response in plant roots, enhancing disease resistance [123]. In strawberries inoculated with *G. mosseae*, the incidence and severity of diseases caused by *Fusarium oxysporum* and *Colletotrichum gloeosporioides* were reduced in the aerial parts and roots. Concurrently, this treatment increased the total polyphenol and ascorbic acid contents [124,125]. AMF also induced “Mycorrhiza-Induced Resistance”, protecting against various pests [126] (Figure 7C).

### 4.4. Limitations of This Study

Although our study adhered to the established principles of bibliometrics and content analysis, there were some limitations. First, we focused on English language articles published within a specific timeframe. While these publications represented over 97% of the retrieved articles, the study was subject to language and temporal constraints. Second, our search strategy aimed to capture various relevant studies by including terms related to fungi, such as BCAs and biofertilisers, while attempting to exclude irrelevant literature using the NOT operator. However, given the inherent limitations of the search algorithms, a few unrelated publications may have been inadvertently included. Third, bibliometric analysis lacks universally accepted standards, and variations in analytical tools and methods can introduce subjectivity into the research process. Despite these limitations, the large dataset analysed in this study offers a comprehensive overview and valuable insights into the current state of research. It also serves as a meaningful guide for future investigations.

## 5. Conclusions

The results of this study highlighted the value of bibliometric techniques in revealing global research trends in fungi, such as BCAs and biofertilisers in the field of biocontrol. According to the survey results, fungal biocontrol has attracted increasing academic attention recently. This research field differs in various countries and regions. China and the USA are the main promoters and leaders of fungal biocontrol and biofertiliser research. This study mainly focused on the core themes of biocontrol, fungi with biocontrol potential, types of diseases solved, and their effects. Among them, EPF, such as *B. bassiana* and *M. anisopliae*, are research hotspots. This article presents several types of fungi that exhibit promise as BCAs and biofertilisers for fungal applications and outlines their mechanisms of action. This study helps to understand the current status of fungi as BCAs and biofertilisers and can be used to guide future research.

## Figures and Tables

**Table 1:** Top 10 most-cited articles published between 1976 and 2024.

No.	Title	TC	TC/Y	Journal	Year	Country	DOI
1	A *Beauveria* phylogeny inferred from nuclear ITS and EF1-alpha sequences: evidence for cryptic diversification and links to *Cordyceps* teleomorphs	1332	66.60	Mycologia	2005	USA	https://doi.org/10.3852/mycologia.97.1.84
2	Persistent negative effects of pesticides on biodiversity and biological control potential on European farmland	898	59.87	Basic Appl. Ecol.	2010	The Netherlands	https://doi.org/10.1016/j.baae.2009.12.001
3	Natural products in crop protection	840	52.50	Bioorg. Med. Chem.	2009	USA	https://doi.org/10.1016/j.bmc.2009.01.046
4	Soil biota and exotic plant invasion	712	33.90	Nature	2004	USA	https://doi.org/10.1038/nature02322
5	*Trichoderma*: The genomics of opportunistic success	617	44.07	Nat. Rev. Microbiol.	2011	Austria	https://doi.org/10.1038/nrmicro2637
6	Mycorrhiza-induced resistance and priming of plant defenses	599	46.08	J. Chem Ecol.	2012	Spain	https://doi.org/10.1007/s10886-012-0134-6
7	Soil biota, ecosystem services and land productivity	574	31.89	Ecol. Econ.	2007	Colombia	https://doi.org/10.1016/j.ecolecon.2007.03.004
8	Twenty years of postharvest biocontrol research: Is it time for a new paradigm?	545	34.06	Postharvest Biol. Technol.	2008	USA	https://doi.org/10.1016/j.postharvbio.2008.11.009
9	Fungal entomopathogens: new insights on their ecology	540	33.75	Fungal Ecol.	2009	USA	https://doi.org/10.1016/j.funeco.2009.05.001
10	The sooty moulds	517	47.00	Fungal Divers.	2014	China	https://doi.org/10.1007/s13225-014-0278-5

TC: total citations; TC/Y: average annual citations since publication.

## Data Availability

The raw data supporting the conclusions of this article will be made available by the authors on request.

## Supplementary Materials

The following supporting information can be downloaded at: https://www.mdpi.com/article/10.3390/jof11020117/s1, Figure S1: Corresponding Author’s Countries (MCP: Number of co-authored papers with authors from other countries; Percentage: International cooperation ratio; SCP: Number of co-authored papers by authors from the same country); Figure S2: Core Sources by Bradford’s Law; Figure S3: Most Relevant Sources (Numbers: Number of publications in the journal).

## Abbreviations

BCAs	Biocontrol agents
WoSCC	Web of Science Core Collection
EPF	Entomopathogenic fungi
USDA	United States Department of Agriculture
AMF	Arbuscular mycorrhizal fungi
CWDEs	Cell-wall-degrading enzymes
