# Supplementary material for: Half-Century Scientometric Analysis: Unveiling the Excellence of Fungi as Biocontrol Agents and Biofertilisers
Source: J Fungi (Basel). 2025 Feb 4;11(2):117. doi: 10.3390/jof11020117 (PMC11856747; doi:10.3390/jof11020117)
Supplement: Supplementary file 1 [file jof-11-00117-s001.zip › jof-3324064-supplementary.pdf]

Supplementary Material

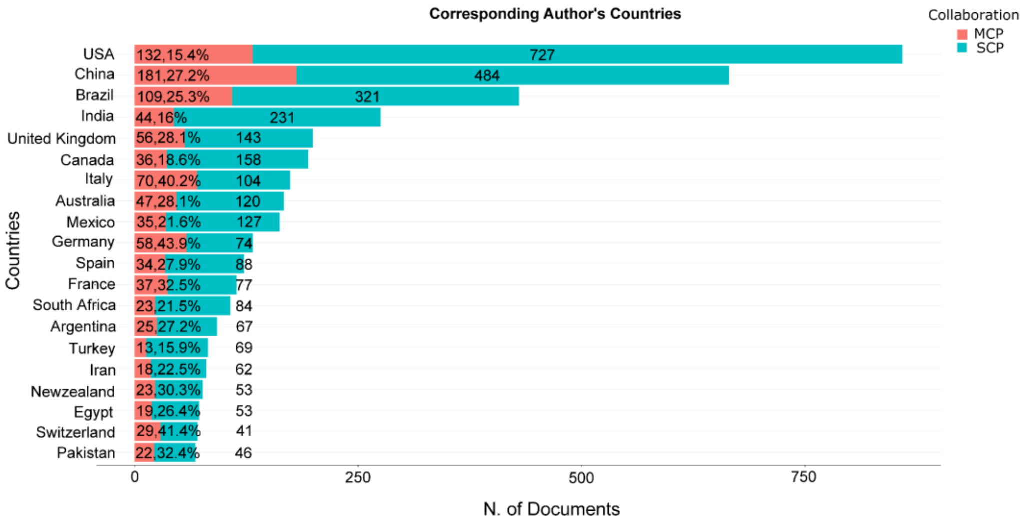

Figure S1. Corresponding Author's Countries (MCP: Number of co-authored papers with authors from other countries; Percentage: International cooperation ratio; SCP: Number of co-authored papers by authors from the same country).

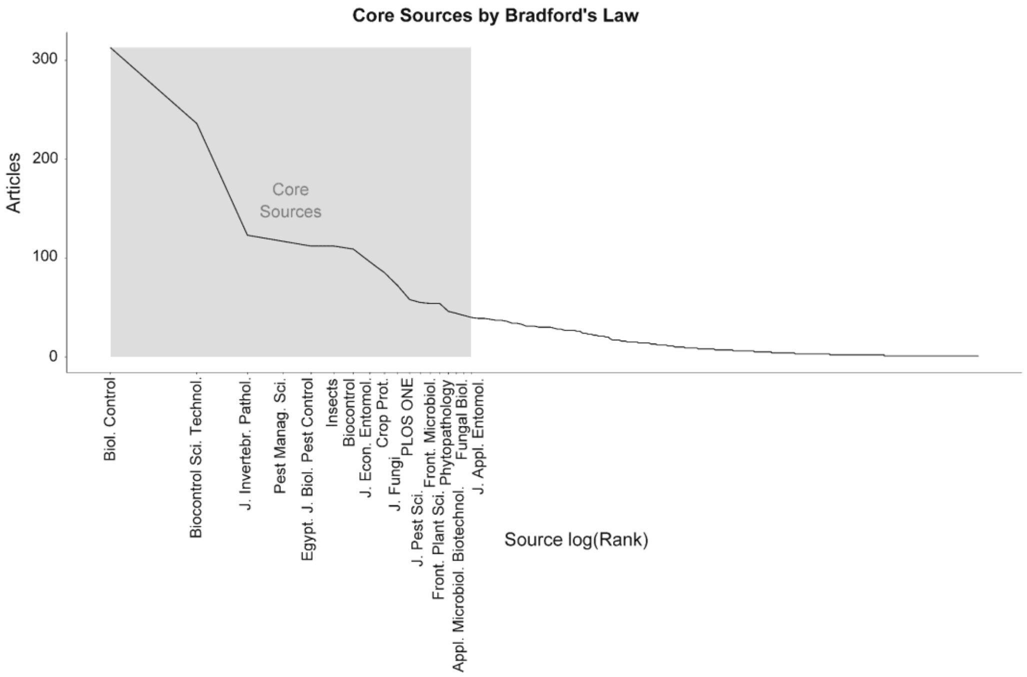

Figure S2. Core Sources by Bradford's Law.

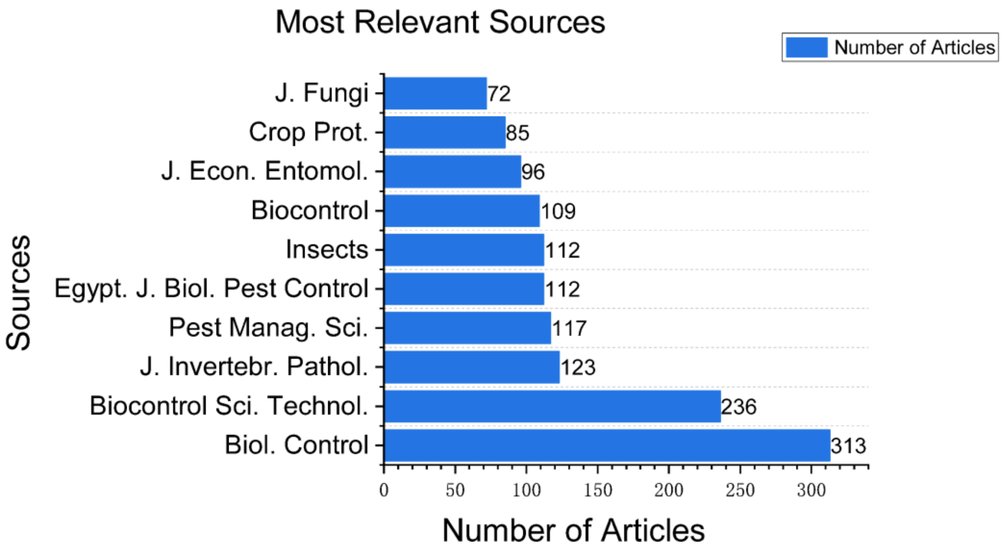

Figure S3. Most Relevant Sources (Numbers: Number of publications in the journal).
